# Nine New Species of *Bugula* Oken (Bryozoa: Cheilostomata) in Brazilian Shallow Waters

**DOI:** 10.1371/journal.pone.0040492

**Published:** 2012-07-12

**Authors:** Leandro M. Vieira, Judith E. Winston, Karin H. Fehlauer-Ale

**Affiliations:** 1 Departamento de Zoologia, Instituto de Biociências, Universidade de São Paulo, São Paulo, São Paulo, Brazil; 2 Laboratório de Sistemática e Evolução de Bryozoa, Centro de Biologia Marinha, Universidade de São Paulo, São Sebastião, São Paudo, Brazil; 3 Virginia Museum of Natural History, Martinsville, Virginia, United States of America; University of Connecticut, United States of America

## Abstract

**Background:**

*Bugula* is a speciose genus of marine bryozoans, represented by both endemic and cosmopolitan species distributed in tropical and temperate waters and important to marine biologists because of the occurrence of many species in harbor and fouling communities, therefore as potential invaders. The southeastern Brazilian coast in the southern Atlantic hosts the highest known diversity of the genus, a status intimately associated with the intensity of collecting efforts.

**Methodology:**

Morphological data based on the examination of living specimens, scanning electron and light microscopic images, and morphometric analyses were used to assess the diversity of *Bugula* along the coastal areas of southern, northeastern, and southeastern Brazil. In this study, morphological species boundaries were based mainly on avicularian characters. For two morphologically very similar species, boundaries are partially supported by 16 S rDNA molecular data.

**Results:**

Nine species are newly described from Brazil, as follows: *Bugula bowiei* n. sp. ( = *Bugula turrita* sensu Marcus, 1937) from the southern, northeastern, and southeastern coasts; *Bugula foliolata* n. sp. ( = *Bugula flabellata* sensu Marcus, 1938), *Bugula guara* n. sp., *Bugula biota* n. sp. and *Bugula ingens* n. sp from the southeastern coast; *Bugula gnoma* n. sp. and *Bugula alba* n. sp. from the northeastern coast; *Bugula rochae* n. sp. ( = *Bugula uniserialis* sensu Marcus, 1937) from the southern coast; and *Bugula migottoi* n. sp., from the southeastern and southern coasts.

**Conclusion:**

The results contribute to the morphological characterization and the knowledge of the species richness of the genus in the southwestern Atlantic (i.e., Brazil), through the description of new species in poorly sampled areas and also on the southeastern coast of that country. Additionally, the taxonomic status of the Brazilian specimens attributed to *B. flabellata*, *B. turrita* and *B. uniserialis* are clarified by detailed studies on zooidal and avicularia morphology.

## Introduction

The widespread cheilostome bryozoan genus *Bugula* Oken, 1815 is ubiquitous in the global ocean, with more than 70 species known [1]. The genus is important for several reasons. Its clearly observable polymorphism, including the unique “bird’s head” avicularia, have made it the textbook example of a bryozoan [2], [3], [4], often the only one most biologists can recognize. One group of *Bugula* species, the *Bugula neritina* (Linnaeus, 1758) complex has also come to the forefront of natural products research because of the medically active macrocyclic lactones called bryostatins found in some of lineages, produced by the endosymbiotic γ-proteobacterium “*Candidatus* Endobugula sertula” [5], [6], [7], [8], [9], [10], [11], [12], [13].

Most important from an environmental standpoint is the fact that many *Bugula* species are found in shallow water and fouling habitats. While many of the species seem to be endemic to a particular region, others, including *Bugula dentata* (Lamouroux, 1816), *B. neritina*, and *Bugula stolonifera* Ryland, 1960 have been classified as widespread and abundant in fouling and harbor communities [7], [14], [15].

In recent years a number of supposedly widespread marine bryozoans have been listed as invasive in different regions [7], [15], [16], [17]. Which of them are really invaders and which are previously unrecognized or misidentified native species is still in question, both in areas where no thorough taxonomic surveys have been carried out, and in regions in which past studies did not have the necessary techniques to distinguish between morphologically similar species. For conservation and management purposes we need to know whether a newly recorded species is an invader, and if so, where it is from, how it arrived, and how likely it is to spread. If it is an endemic species, especially one having a very small geographic range, it may require protection of some kind. In this context, the correct identification of species is the key requirement to adequately access aspects of biodiversity conservation and its appropriate use [18]. However, taxonomic surveys are not well advanced for many taxa and regions in general [18], as is the case for *Bugula* along most of the Brazilian coast.

Recent studies comparing the bryozoan fauna among different localities in Brazil and the Atlantic coast of the USA have revealed an unexpectedly high diversity of species in Brazilian waters, and also have helped resolve the taxonomic identity of putatively widespread taxa [19]. The southeastern Brazilian coast is the best-studied region for species of *Bugula* thanks to work carried out by Marcus [20], [21], [22], and Ramalho *et al.* [23]. The absence of studies on *Bugula* in poorly sampled regions [10] motivated us to focus our collecting efforts in areas other than the southeastern coast, with the aim of finding rare and undescribed species. These efforts allowed us to describe nine new species of *Bugula* collected in additional localities along the northeastern, southern and southeastern Brazilian coast, increasing the total number of *Bugula* species recorded from these regions to 14.

## Materials and Methods

### Collection of Specimens and Morphological Examination

**Figure 1:**
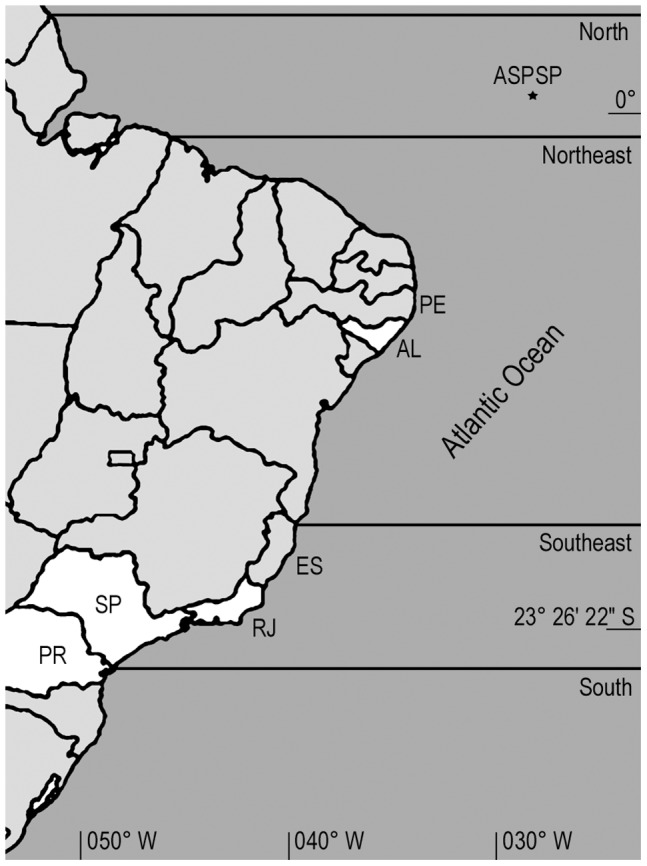
Map of sampling area (in white). Map of sampling area in Brazilian coast (in white).

All necessary permits were obtained for the described field studies (collecting permit numbers 10186 and 19936 SISBIO/Instituto Chico Mendes de Conservação da Biodiversidade) and the reported localities do not include protected areas. No statement from an ethics committee was necessary and the field studies did not involve endangered or protected species. The specimens were collected from intertidal and subtidal localities along the Brazilian coast (Figure 1; Table S1). For comparative purposes, *Bugula turrita* (Desor, 1848) from Woods Hole (NW Atlantic, USA; type locality), and *Bugula fulva* Ryland, 1960 from Wales (NE Atlantic, UK; type locality) were also collected in the intertidal zone. Living colonies were examined and, whenever possible, representative specimens of each species were photographed using a digital camera mounted on a stereo microscope. The tissues were then preserved in 92–99% ETOH. Some preserved specimens were selected for critical-point drying, after dehydration to 100% ethanol in a graded series of ethanol-water. The samples were mounted on stubs and coated with a gold-palladium alloy for observation by scanning electron microscopy (SEM) in JEOL 6460LV and Zeiss LEO 440. Measurements were made with a Zeiss SV-11 stereo microscope with a ocular micrometer. Avicularia of some fixed specimens were observed and photographed using a digital camera mounted on a light microscope. The material studied was deposited in the bryozoan collection at the Museu de Zoologia da Universidade de São Paulo, Brazil (MZUSP), and at the Setor de Comunidades Bentônicas da Universidade Federal de Alagoas, Brazil (UFAL). Additional comparative specimens were examined in the bryozoan collection at the Natural History Museum, London, UK (NHMUK).

The abbreviations used for the Brazilian localities (Figure 1) are: ASPSP, Saint Peter and Saint Paul Archipelago; PE, Pernambuco; AL, Alagoas; ES, Espírito Santo; RJ, Rio de Janeiro; SP, São Paulo; PR, Paraná.

### DNA Analyses

Total genomic DNA was extracted from representative specimens of two new species (*Bugula ingens* n. sp. and *Bugula rochae* n. sp.) with the DNeasy® Blood & Tissue Kit (QIAGEN). Approximately 50 clean zooids per colony were used for each extraction. The primer set primer 1+ primer 2 [24] was used for PCR and sequencing of ∼500 bp of the mitochondrial gene 16S rDNA, commencing approximately at a position homologous to position 3257 of the *B. neritina* complete mitochondrial genome (GenBank AY690838). PCRs were carried out in 25 µl volumes using illustra PuReTaq Ready-To-Go™ PCR Beads (GE Healthcare). Cycling conditions for the amplification were: 94°C, 5′ - 35×: 94°C, 30′′; 55°C, 30′′; 72°C, 45′′ - 72°C, 3′. PCR products were cleaned with illustra GFX™ PCR DNA and Gel Band Purification Kit (GE Healthcare). Sequencing in both directions was performed with a BigDye®Terminator v3.1 Cycle Sequencing Kit (Applied Biosystems) and an ABI PRISM® 3100 Genetic Analyzer. Complementary strands were combined and edited with CodonCode Aligner (CodonCode Corporation), and compiled using Geneious Pro v5.3.6 [25]. We searched GenBank for sequences similar to ours using BLAST [http://blast.ncbi.nlm.nih.gov/], and deposited our unique sequences in GenBank and in the Barcode of Life Database System (BOLD) [26], as part of the Brazilian Barcode of Life (BrBOL) initiative (http://www.brbol.org/content/brbol-brazilian-barcode-life; Pj2, Brazilian Initiative for Molecular Identification of Marine Organisms). The sequences generated by us and six sequences of *Bugula* retrieved from GenBank (*B. dentata*, AY633933; *Bugula pacifica* Robertson, 1905, AY633941; *Bugula simplex* Hincks, 1886, AY633942; *B. stolonifera*, AY633944; *Bugula turbinata* Alder, 1857, AY633946; *B. neritina*, AY633935) were aligned in MAFFT [27] using default settings, and verified by eye in Geneious Pro. Pairwise sequence divergence were calculated using the Kimura-2-parameter model available in MEGA 5.0 [28].

### Nomenclatural Acts

The electronic version of this document does not represent a published work according to the International Code of Zoological Nomenclature (ICZN), and hence the nomenclatural acts contained in the electronic version are not available under that Code from the electronic edition. Therefore, a separate edition of this document was produced by a method that assures numerous identical and durable copies, and those copies were simultaneously obtainable (from the publication date noted on the first page of this article) for the purpose of providing a public and permanent scientific record, in accordance with Article 8.1 of the Code. The separate print-only edition is available on request from PLoS by sending a request to PLoS ONE, Public Library of Science, 1160 Battery Street, Suite 100, San Francisco, CA 94111, USA along with a check for $10 (to cover printing and postage) payable to “Public Library of Science”.

In addition, this published work and the nomenclatural acts it contains have been registered in ZooBank, the proposed online registration system for the ICZN. The ZooBank LSIDs (Life Science Identifiers) can be resolved and the associated information viewed through any standard web browser by appending the LSID to the prefix “http://zoobank.org/”. The LSID for this publication is: (urn:lsid:zoobank.org:pub:1C36F2BA-F9EA-46B8-BD9C-EF6F6B0D1010).

## Results

### Systematic Account

**Figure 2:**
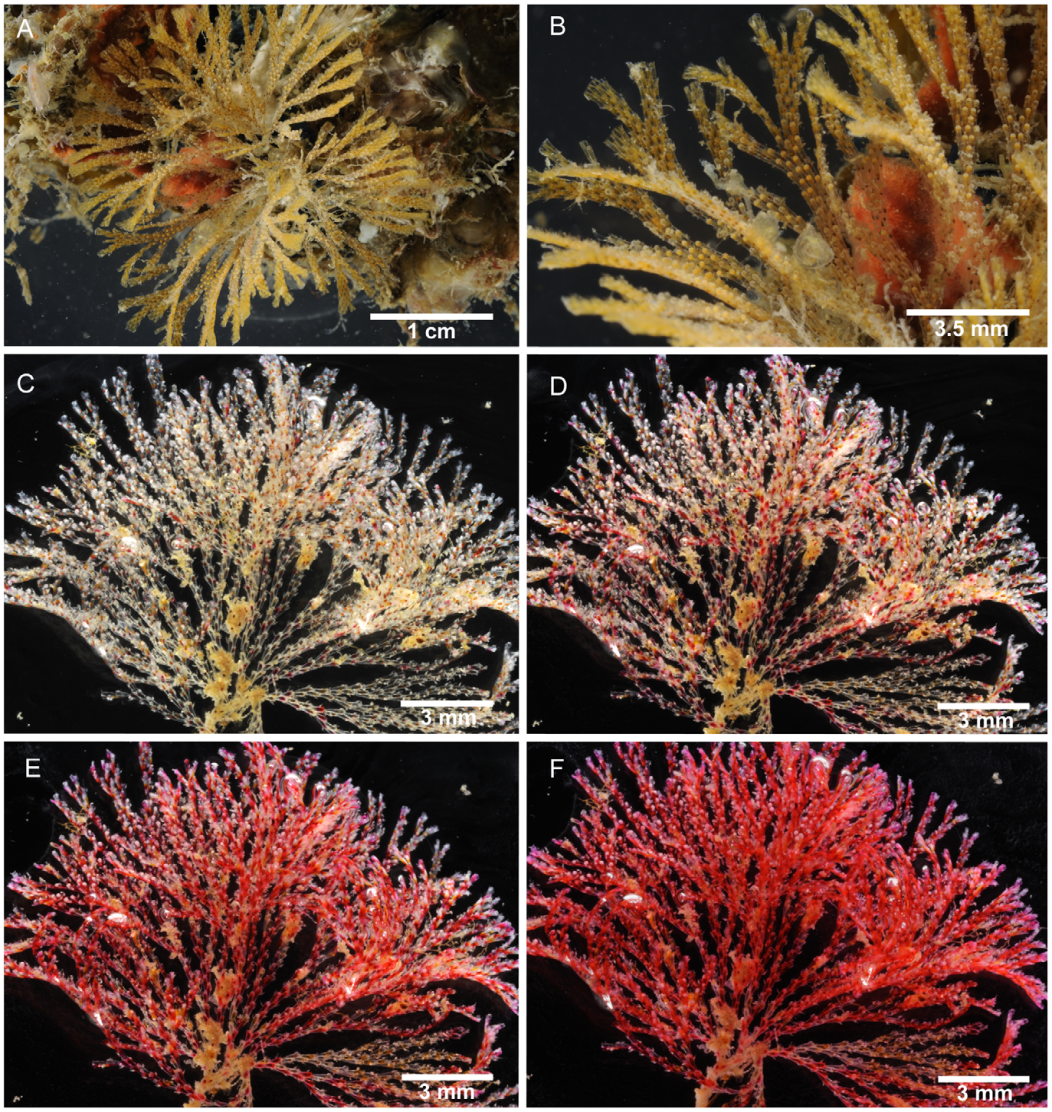
Light microscope images of *Bugula foliolata* n. sp. and *Bugula guara* n. sp. A–B, *Bugula foliolata* n. sp., MZUSP 570, Holotype, Rio de Janeiro, Brazil. C–F, *Bugula guara* n. sp., MZUSP 591, Holotype, Rio de Janeiro, Brazil, showing the fixation process in alcohol 92.2%, (C) after 10 seconds, (D) after 30 seconds, (E) after 60 seconds and (F) after 90 seconds.

**Figure 3:**
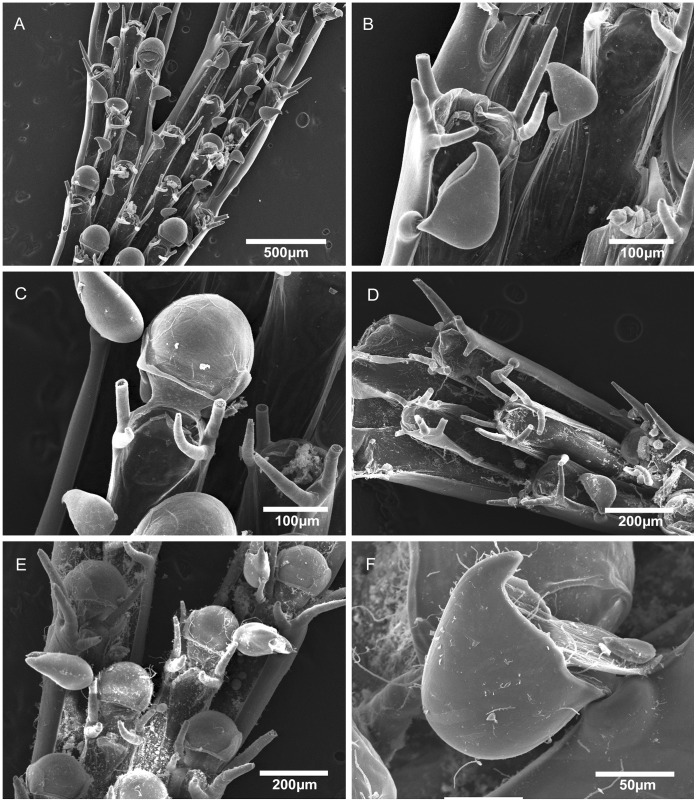
Scanning electron micrographs of *Bugula foliolata* n. sp. from Brazil and *Bugula fulva* from UK. A–D, *Bugula foliolata* n. sp., MZUSP 570, Holotype, Rio de Janeiro, Brazil, (A) multiserial branch, (B) close-up of avicularia in marginal and internal zooids, (C) ovicelled zooid, (D) distal end of the branch, showing the zooids with six oral spines. E–F, *Bugula fulva* Ryland, MZUSP 640, UK, (E) axis of the branch, (F) close-up of avicularium.

**Bugula foliolata** Vieira, Winston & Fehlauer-Ale **n. sp.**

urn:lsid:zoobank.org:act:8B45FAF0-D47E-42D7-81F3-38F8A9C9B015.

(Figures 2A–B, 3A–D; Table 1).

**Table 1:** Measurements (mm; n = 15) of *Bugula* species from Brazilian coast and the NW Atlantic *B. turrita*.

	*Bugula foliolata* n. sp.	*Bugula bowiei* n. sp.	*Bugula guara* n. sp.	*Bugula turrita*
Zooid length				
Range	0.598–0.736	0.432–0.644	0.405–0.745	0.460–0.718
Mean (St. dev.)	0.641 (0.042)	0.516 (0.074)	0.555 (0.089)	0.554 (0.083)
Zooid width				
Range	0.138–0.166	0.138–0.184	0.110–0.138	0.138–0.212
Mean (St. dev.)	0.150 (0.008)	0.168 (0.016)	0.123 (0.011)	0.157 (0.021)
Frontal membrane				
Range	0.534–0.681	0.368–0.423	0.322–0.460	0.414–0.460
Mean (St. dev.)	0.600 (0.040)	0.389 (0.017)	0.419 (0.044)	0.438 (0.014)
Avicularia length				
Range	0.138–0.166	0.147–0.179	0.161–0.185	0.101–0.129
Mean (St. dev.)	0.156 (0.010)	0.162 (0.010)	0.172 (0.008)	0.116 (0.010)
Avicularia width				
Range	0.078–0.101	0.069–0.087	0.051–0.064	0.055–0.069
Mean (St. dev.)	0.093 (0.007)	0.076 (0.006)	0.056 (0.004)	0.060 (0.005)
Avicularia length(2)				
Range	0.083–0.115	–	–	–
Mean (St. dev.)	0.103 (0.009)	–	–	–
Avicularia width(2)				
Range	0.055–0.064	–	–	–
Mean (St. dev.)	0.060 (0.003)	–	–	–
Ovicell width				
Range	0.143–0.161	0.115–0.138	0.110–0.124	0.147–0.179
Mean (St. dev.)	0.153 (0.006)	0.126 (0.008)	0.117 (0.008)	0.164 (0.013)

*Bugula ditrupae*: Marcus, 1937∶69, plate 14, figure 36 [20]. Non *Bugula ditrupae* Busk, 1858 [29].

*Bugula flabellate*: Marcus, 1938∶27 (part), plate 5, figure 13a [21] (non plate 6, figure 13b;  = *Bugula carvalhoi* Marcus, 1949 [22]). Non *Bugula flabellata* (Thompson in Gray, 1848) [30].

**Material examined. Holotype**: MZUSP 570, Angra dos Reis, RJ, Brazil. **Additional material examined**: MZUSP 008, *Bugula ditrupae* Busk, E. Marcus det.; MZUSP 077, MZUSP 078, MZUSP 080, Caraguatatuba, SP, Brazil. **Comparative specimens**: MZUSP 639–640, *Bugula fulva* Ryland, 1960, Wales, UK.

#### Type locality

Sitio Forte Bay, Ilha Grande, Angra dos Reis, RJ, Brazil: 23°07′00′′S, 044°16′50′′W.

#### Diagnosis

Colonies form flat fans, with branches 2–8 zooids wide; zooids narrower and longer than those of *B. fulva* and *B. flabellata*, two other species with strap-like branches; external zooids with three outer and two inner spines; the internal zooids with two or three pairs of spines; avicularia longer and more slender than those of *B. fulva*, and the rostrum distally rounded.

#### Description

Colonies form flat fans of strap-like branches, a few centimetres long, reddish-brown in colour (Figures 2A–B). Zooids arranged in quincux, with branches 2–8 zooids wide. Zooids elongate, rectangular, four times longer than wide, with the frontal membrane occupying almost the entire frontal area. Distal corners of zooids sharply pointed. Zooids at the outer edge of branches have thicker outer lateral and distal walls and bear three outer and two inner distal spines. Inner zooids usually with two outer and two inner spines; terminal zooids on branches with three outer and three inner spines. Lower spines curve toward the midline of the zooid, arching over the distal end of the frontal membrane. Pedunculate avicularia dimorphic (length to width ratio: small avicularia 1.5–2∶1, large avicularia 1.4–1.7∶1), almost subtriangular in profile, with a round body and long rostrum, flat abfrontal surfaces with no indentation between body and distal end of the rostrum whose tip curves downward to a blunt-pointed hook; avicularia positioned about 1/4 of the way down outer lateral walls of zooids attached to peduncle by a short pointed projection; marginal avicularia on outer zooids larger than those on inner zooids, their ventral rims biconvex, raised at center edge, where mandible is hinged, small avicularia with a straight ventral margin. Ovicells centered in midline, globular, distinct. Ancestrula often obscured by rhizoids, vase-shaped, having a circular, terminal frontal membrane, with three pairs of spines.

#### Etymology

From the Latin noun *folium*, leaf, plus the Latin adjective *latus*, broad, in allusion to the wide branches of the colony.

#### Remarks

Two multiserial species of *Bugula* were previously reported along the Brazilian coast: *Bugula flabellata* (Thompson in Gray, 1848) and *Bugula ditrupae* Busk, 1858. *Bugula flabellata* is a cold water species mainly known from Britain and northern Europe, but apparently also a successful fouling bryozoan, documented as introduced on the southern Australia and New Zealand coasts [31].

Ryland [32] noted confusion in the taxonomic literature between *B. flabellata* and four other species: *Bugula aquilostris* Ryland, 1960, *B. fulva*, *B. ditrupae* and *Bugula philippsae* Harmer, 1926. The number of zooids in branches, position and shape of avicularia, number of spines and shape of ovicells are distinguishing characters among those species. Both *B. flabellata* and *B. ditrupae* from Brazil were lately reassigned as *B. fulva* and *B. aquilirostris*, respectively [32], and both were posteriorly combined into *B. fulva* [33]. The North Carolina specimens identified as *B. fulva* are in the middle of the range of variation between *B. fulva* and *B. aquilirostris* [34], a characteristic that we also observed in the Brazilian specimens: external zooids with three outer and two inner spines, internal zooids with two inner and two outer zooids (except in zooids in the distal part of the colony, which have three outer and three inner spines). Further comparison between specimens of *B. fulva* from North Carolina [34] and from Europe is required. Hayward and McKinney [35] suggested that *B. fulva* is a fouling species that occurs patchily in southern Britain and in the Mediterranean and may have an exotic origin. Although Maturo [36] describes it as occurring on the continental shelf of the eastern United States both North and South of Cape Hatteras, an extensive study of intertidal and subtidal localities from Maine to Virginia, found it only in the Gulf of Maine [37]. Our comparison between the Brazilian specimens and colonies of *B. fulva* from UK (Figures 3E–F) revealed differences in zooidal size, position and shape of avicularia, which led us to describe the colonies from Brazil as *Bugula foliolata* n. sp.

*Bugula foliolata* n. sp. has narrower and longer zooids than *B. fulva* and *B. flabellata*. The avicularium is longer and more slender than that of *B. fulva*, and the rostrum is distally rounded, not a straight backed and abruptly hooked chamber as in *B. flabellata* [32], [38]. Marcus [21] reported two distinct morphotypes from São Paulo under the name *B. flabellata*; his figured specimen from Santos (figure 13A) [21] is here reassigned to *Bugula foliolata* n. sp., while the specimens with fewer spines from the locality of Ilha das Palmas (figure 13B) [21] were later referred by him to *Bugula carvalhoi* Marcus, 1949 [22].

#### Distribution (present study)

Brazil: Angra dos Reis, RJ (Table S1); São Sebastião, SP (Table S1); on stones, shells and other bryozoans at 7–20 meters depth.

### Species of the *Bugula turrita* Group

**Figure 4:**
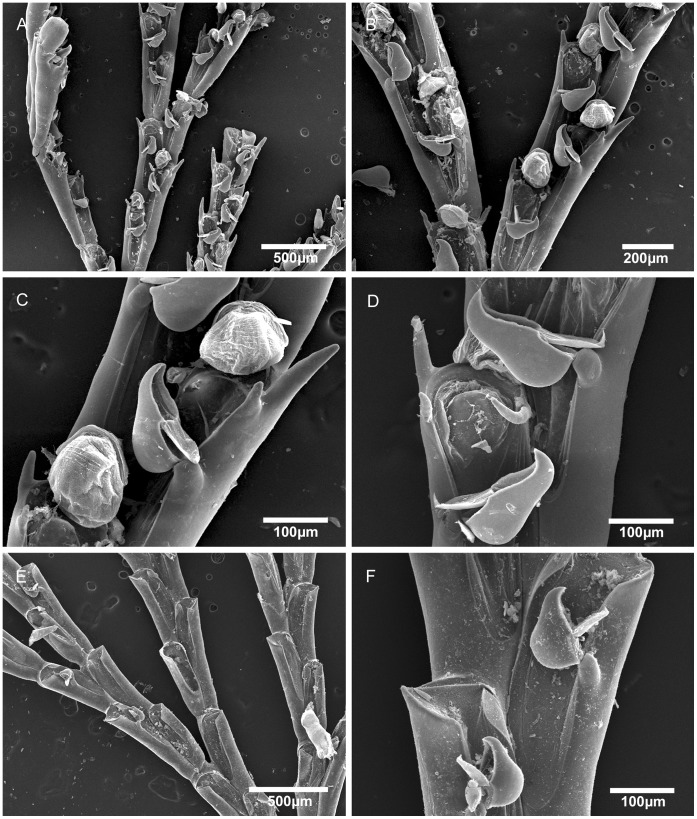
Scanning electron micrographs of *Bugula bowiei* n. sp. from Brazil and *Bugula turrita* from USA. A–D, *Bugula bowiei* n. sp., MZUSP 571, Holotype, Paraná, Brazil, (A) branches, (B) bifurcation, (C) close-up of ovicelled zooids, (D) close-up of avicularia and oral spines. E–F, *Bugula turrita* Oken, MZUSP 638, Woods Hole, USA, (E) branches, (F) close-up of avicularium.

Specimens of *B. turrita* from Woods Hole (Massachusetts, USA; type locality) and Brazil share similar morphological characters such as the position of avicularia, the number of zooidal spines, and branching pattern (Figures 4A–F). In Brazil, *B. turrita* was previously recorded from the southern and southeastern coasts [20], [39], [40]. However, Maturo [34] noted differences between the Brazilian material and specimens from Florida (USA) attributed to *B. turrita* (e.g. presence of spines in ancestrula), as well as the presence of some closely-related taxa on the Atlantic coasts of the USA and the Caribbean, morphologically distinguished by the number of spines, position and shape of avicularia.

**Figure 5:**
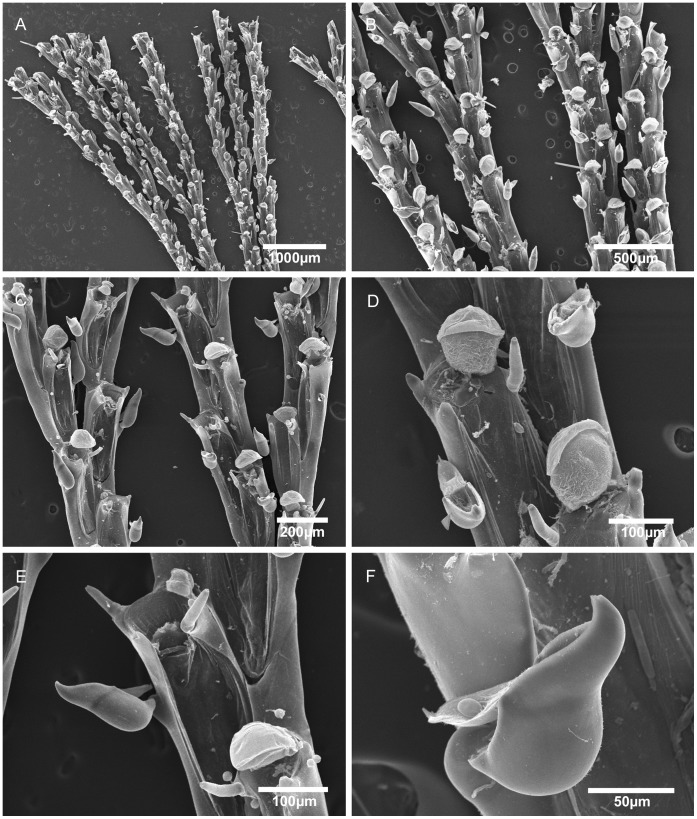
Scanning electron micrographs of *Bugula guara* n. sp. *Bugula guara* n. sp., MZUSP 591, Holotype, Rio de Janeiro, Brazil, (A) colony, (B) close-up of branches with ovicelled zooids, (C) bifurcation, (D) close-up of ovicelled zooids, (E) close-up of zooid, showing three oral spines and marginal avicularium, (F) close-up of avicularium.

Among the Brazilian specimens we found two distinct morphotypes initially attributed to *B. turrita*: one characterized by robust colonies as described by Marcus [20], recorded here from Maceió (AL), São Sebastião (SP) and Ilha do Mel (PR) (Figures 4A–D); the other includes specimens with slender colonies and longer avicularia, only found in Ilha Grande (RJ) (Figures 2C–F, 5A–F). Specimens of *B. turrita* from the northeastern coast of the USA (Figures 4E–F) are distinct from both Brazilian morphotypes in having very short spines and smaller avicularia. Based on those differences, we describe the two Brazilian forms as *Bugula bowiei* n. sp. and *Bugula guara* n. sp., respectively.

***Bugula bowiei*** Vieira, Winston & Fehlauer-Ale **n. sp.**

urn:lsid:zoobank.org:act:8E6F94C3-C434-443E-83BA-5C75055DC292.

(Figures 4A–D; Table 1).

*Bugula turrita*: Marcus, 1937∶68, plate 14, figure 35 [20]. Non *Cellularia turrita* Desor, 1848 [41].

?*Acamarchis brasiliensis* d’Orbigny, 1841∶10, plate 3, figures 5–8 [42].

**Figure 6 pone-0040492-g006:**
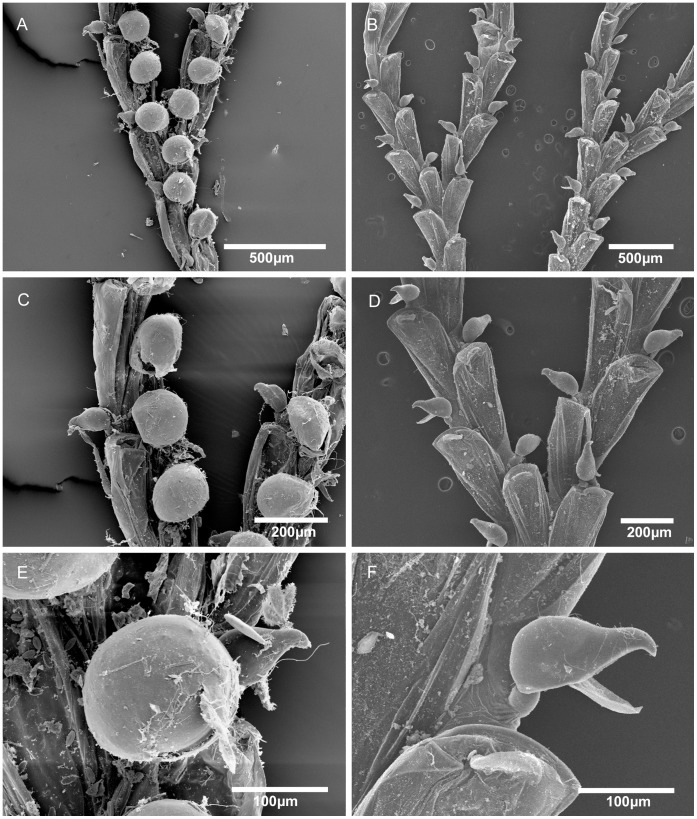
Scanning electron micrographs of *Bugula alba* n. sp. and *Bugula migottoi* n. sp. A,C,E, *Bugula alba* n. sp., MZUSP 600, Paratype, Alagoas, Brazil, (A) branches, (C) bifurcation, (E) close-up of avicularium. B,D,F, *Bugula migottoi* n. sp., MZUSP 602, Paratype, Paraná, Brazil, (B) branches, (D) bifurcation, (F) close-up of avicularium.

**Figure 7 pone-0040492-g007:**
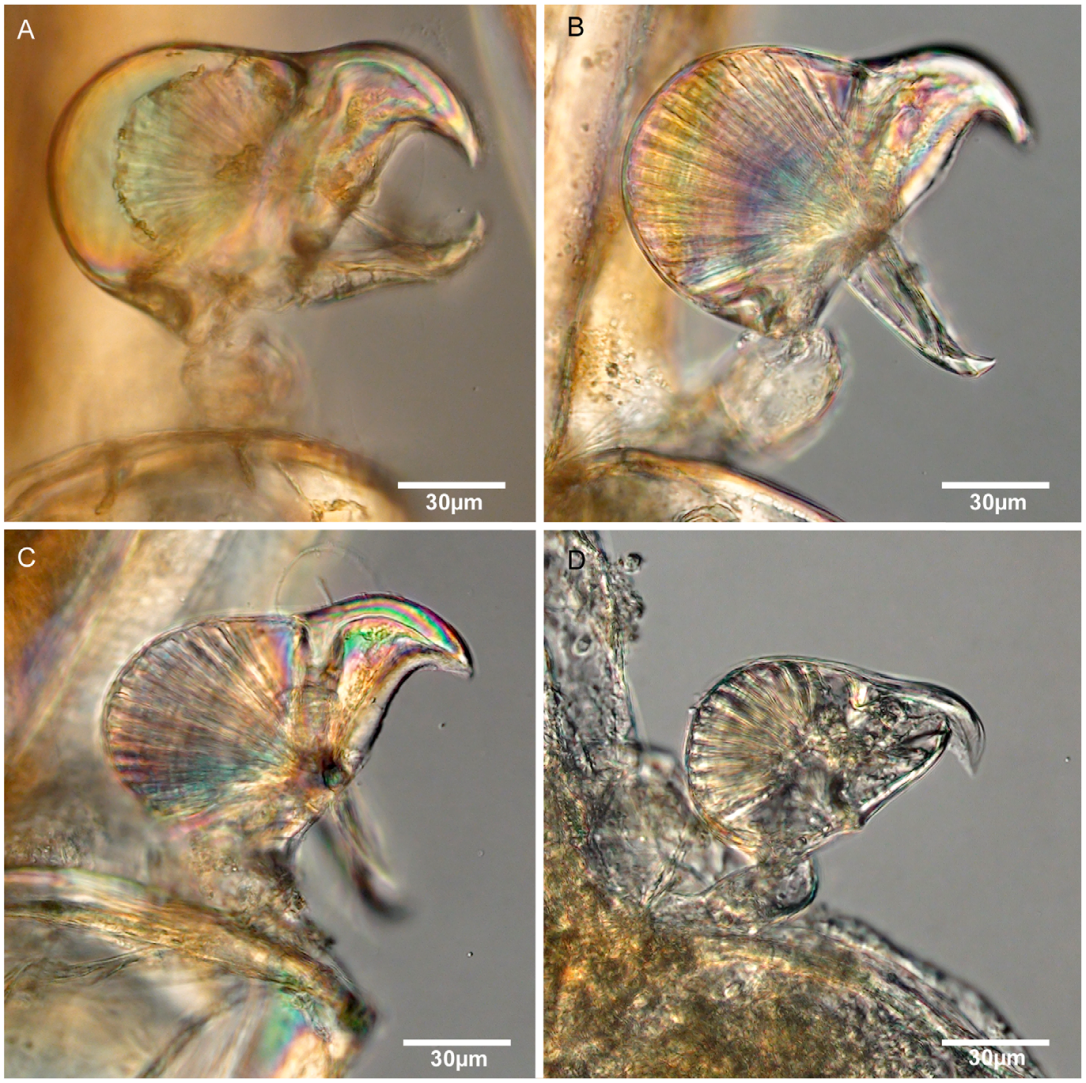
Light microscope images of avicularia of Brazilian species of *Bugula uniserialis* group. Close-up of avicularia, (A) *Bugula biota* n. sp., MZUSP 618, Paratype, São Paulo, Brazil, (B) *Bugula ingens* n. sp., MZUSP 627, Paratype, Rio de Janeiro, Brazil, (C) *Bugula rochae* n. sp., MZUSP 631, Paratype, Paraná, Brazil, (D) *Bugula gnoma* n. sp., MZUSP 623, Paratype, Alagoas, Brazil.

**Figure 8:**
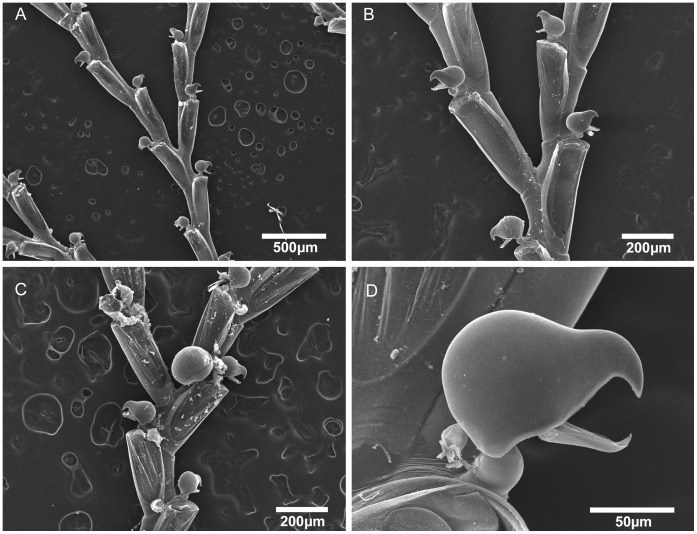
Scanning electron micrographs of *Bugula biota* n. sp. *Bugula biota* n. sp., MZUSP 618, Paratype, São Paulo, Brazil, (A) colony, (B) bifurcation, (C) close-up of bifurcation and ovicelled zooids, (D) close-up of avicularium.

**Material examined. Holotype**: MZUSP 571, Ilha do Mel, PR, Brazil. **Paratypes**: MZUSP 572–573, Ilha do Mel, PR, Brazil. Additional material: MZUSP 574–578, Maceió, AL, Brazil; MZUSP 579–585, Ilha do Mel, Brazil; MZUSP 586–590, São Sebastião, SP, Brazil. **Comparative specimen**: MZUSP 638, *Bugula turrita* (Desor), Woods Hole, USA.

#### Type locality

Encantadas, Ilha do Mel, PR, Brazil: 25°34′26′′S, 048°19′07′′W.

#### Diagnosis

Erect biserial branching colonies with bifurcation of type 3 of Harmer [43], zooids with two outer and one inner distal spines, monomorphic avicularia on all zooids, placed at the midpoint of the outer side of the frontal membrane; globular ovicells slightly shifted toward inner margin.

#### Description

Colony erect, biserial, yellow-brownish in colour, consisting of robust primary and secondary branches developing in spiralling fans or whorls, attached to substrata by long radicles; bifurcation type 3 [43]. Zooids elongate, tapering proximally; zooids at bifurcations longer (about 0.60 mm) than others (about 0.52 mm). Frontal membrane occupies most of zooidal frontal length, its plane tilted toward the median axis of the branch. Zooids with two outer and one inner distal spines; outer distal corner of zooid is a very long robust spine, the spine below it shorter; inner side with one spine usually bent forward slightly. Pedunculate avicularia monomorphic, elongate with a shallow depression separating body and rostrum (sway-backed), length to width ratio 1.8–2.4∶1, rostrum curved, pointed tip; lateral edges of avicularium in profile slightly raised at point where mandible is hinged, then curving to distal tip of rostrum, attached laterally at the middle of opesial length; head elongate and rostrum down-curved distally. Ovicells deep, rounded caps, globular, somewhat irregular and rugose, slightly shifted toward inner margin, sometimes with calcified area at its distal edge, about 0.12 mm wide, attached at distal ends of maternal zooids. Ancestrula vase-shaped, having a circular, terminal frontal membrane, with some well-spaced spines; aberrant zooids often present along the branches, uniserial, ancestrula-shaped. Colony attached by some rootlets (rhizoids) to the substrate.

#### Etymology

Named after David Bowie, British popular musician (1947–) and third author’s favourite artist.

#### Remarks

Marcus [20] recorded this species from São Paulo under the name *B. turrita*. *Bugula turrita* seems to be limited to the western Atlantic in the northern hemisphere; it is characterized by yellow-orange colonies, the frontal membrane occupying about two-thirds of the frontal area, and small avicularia (Figures 4E–F; Table 1), shorter than those of *B. bowiei* n. sp (Figure 4D). *Bugula guara* n. sp. from Rio de Janeiro is distinct from *B. bowiei* n. sp. through the presence of red pigments inside the polypides in living colonies (colonies seem to get more reddish with increasing immersion time in alcohol, but become white after few minutes; Figures 2C–F), the shape of the colony and the shape of avicularia, which are more slender in *B. guara* n. sp. than in either *B. turrita* or *B. bowiei* n. sp.

According to Hastings [44], the zooids of *B. turrita* are quite similar to *Bugula hyadesi* Jullien, 1888, also described from the Atlantic [45]. *Bugula hyadesi* differs from *B. turrita* and *B. bowiei* n. sp. by the shorter cap-like ovicells and larger avicularia, attached at the outer proximal corner of the opesia. D’Orbigny also described *Acamarchis brasiliensis* d’Orbigny, 1841, from Rio de Janeiro, Brazil, but as his original description was based on specimens without ovicells and avicularia [42], his species could be *B. hyadesi*, *B. bowiei* n. sp., or *B. guara* n. sp, and its true identity remains uncertain.

#### Distribution (present study)

Brazil: Maceió, AL (Table S1); São Sebastião, SP (Table S1); Ilha do Mel, PR (Table S1); found on panels, algae, stones and other bryozoans at 0–3 meters depth.

***Bugula guara*** Vieira, Winston & Fehlauer-Ale **n. sp.**

urn:lsid:zoobank.org:act:F955AD64-7547-4189-B299-839BA8F1FBCE.

(Figures 2C–F, 5A–F; Table 1).

?*Acamarchis brasiliensis* d’Orbigny, 1841∶10, plate 3, figures. 5–8 [42].

**Material examined. Holotype**: MZUSP 591, Ilha Grande, RJ, Brazil. **Paratypes**: MZUSP 592–597, Ilha Grande, RJ, Brazil.

#### Type locality

Laje Branca, Ilha Grande, Angra dos Reis, RJ, Brazil: 23°08′15′′S, 044°20′49′′W.

#### Diagnosis

Erect biserial colonies with bifurcation type 3; zooids with two outer and one inner distal spines; avicularia more slender than those of *B. bowiei* n. sp. (ca. 0.17 mm long and 0.06 mm wide) attached about 1/3 of the length down of the outer side of the frontal membrane; ovicell well calcified at its distal edge.

#### Description

Colony erect, translucent white in colour in life, at first turning a reddish colour with increasing immersion in alcohol (Figures 2C–F); colonies are translucent white after 5 minutes or more of immersion in alcohol. Colonies with delicate primary and secondary branches developing in whorls, attached to substrata by radicles; bifurcation of type 3 [43]. Zooids elongate, almost rectangular; outer zooids of the bifurcation longer than others (about 0.75 mm). Frontal membrane with U-shaped proximal edge, occupying almost all of the frontal area, directed toward the median axis of the branch. Outer distal corner has a slender spine with a shorter spine below it; inner side with a longer spine bent forward slightly. Pedunculate avicularia monomorphic, body rounded proximally, shallow V-shaped indentation between abfrontal body surface and rostrum, much longer than wide, length to width ratio 2.6–3∶1; attached on the lateral margin about 1/3 the length of the zooid from the distal end; head longer than wide, with long rostrum down-curved distally and in profile with a raised hinge-point; a convex scalloped edge on body side, and a longer asymmetrically curved edge leading to hooked tip of rostrum. Ovicells deep, rounded caps, with calcified area occupying to 1/3 of its distal end, attached in zooidal midline. Ancestrula elongate, obscured by basal rhizoids.

#### Etymology

Epithet, a noun in apposition, named for the ‘guará’, *Eudocimus rubber* (Linnaeus, 1758), the scarlet ibis, a bright red tropical bird common in Brazil, in allusion to the red pigment of the polypides that are lost after their fixation in ethanol.

#### Remarks

*Bugula guara* n. sp. has a type 3 branching pattern [43], as previously described for other Atlantic species such as *B. bowiei* n. sp., *Bugula marcusi* Maturo, 1966, *Bugula microoecia* Osburn, 1914, *Bugula rylandi* Maturo, 1966, and *B. turrita* [34], [46]. These species differ from *B. guara* n. sp. in the zooid size, ovicell shape, and the shape and position of the avicularia. *Bugula marcusi* is similar to *B. guara* n. sp. in branching pattern and zooid size, but differs in the position of the elongate avicularia, which are attached halfway down or lower on the outer frontal margin.

#### Distribution (present study)

Brazil: Ilha Grande, RJ (Table S1); found on algae, stones and other bryozoans at 5–15 meters depth.

### Species of the *Bugula Minima* Group

Recently, Winston & Woollacott [8] redescribed nine species of reddish *Bugula*, some of which were previously recorded as *Bugula minima* Waters, 1909 in Pacific and Atlantic waters. Western Atlantic material previously identified as *Bugula minima* belongs to *Bugula miniatella* Winston & Woollacott 2008, a species characterized by small zooid and colony size, as well as early sexual reproduction; it is more similar to *Bugula crosslandi* Hastings, 1939 and to material from the eastern Pacific also identified by R. Osburn [47] and A. Hastings [48] as either *B. minima* or *B. crosslandi*, and perhaps may comprise a species complex. The *B. minima* of Waters [49], originally described from the Red Sea, is a much larger and more robust species, with affinities and similarities to other *Bugula* from the Indo-Pacific, which may make up another species complex [8].

***Bugula alba*** Vieira, Winston & Fehlauer-Ale **n. sp.**

urn:lsid:zoobank.org:act:08F431D3-535E-4E3D-827D-D35FE3EE38F0.

(Figures 6A, C, E; Table 2).

**Table 2:** Measurements (mm; n = 15) of *Bugula minima* “group” found in Atlantic coast.

	*Bugula alba* n. sp.	*Bugula migottoi* n. sp.	*Bugula miniatella*
Zooid length			
Range	0.414–0.570	0.506–0.681	0.53–0.66
Mean (St. dev.)	0.493 (0.051)	0.585 (0.049)	0.58 (0.05)
Zooid width			
Range	0.138–0.166	0.156–0.202	0.15–0.19
Mean (St. dev.)	0.147 (0.012)	0.181 (0.014)	0.18 (0.02)
Frontal membrane			
Range	0.322–0.350	0.377–0.478	–
Mean (St. dev.)	0.331 (0.011)	0.414 (0.028)	–
Avicularia length			
Range	0.110–0.133	0.152–0.175	0.19–0.25
Mean (St. dev.)	0.123 (0.009)	0.162 (0.008)	0.22 (0.02)
Avicularia width			
Range	0.055–0.069	0.069–0.083	0.08–0.10
Mean (St. dev.)	0.064 (0.005)	0.076 (0.005)	0.09 (0.01)
Ovicell length			
Range	–	–	0.171–0.190
Mean (St. dev.)	0.160	–	0.19 (0.001)

Measurements of *Bugula miniatella* from Winston & Woollacott (2008).

**Material examined. Holotype**: MZUSP 598, Maceió, AL, Brazil. **Paratypes**: MZUSP 599–600, Maceió, AL, Brazil.

#### Type locality

Riacho Doce, Maceió, AL, Brazil: 9°34′42′′S, 035°39′19′′W.

#### Diagnosis

Erect colonies, delicate, with branches curving at the tips; small zooids (ca. 0.49 mm long and 0.15 mm wide) and small avicularia (ca. 0.12 mm long) attached at the outer side of the proximal end of zooids, smaller than those of *B. miniatella* and *Bugula migottoi* n. sp.

#### Description

Colony erect, very small, delicate, with biserial branches curving at the tips of the colony; living colonies translucent, white to pale yellowish in colour. Zooids elongate, almost rectangular, slightly wider at distal end; frontal membrane occupying almost the entire frontal surface. Distal spines absent; outer distal corner pointed, inner distal corner rounded to slightly pointed. Avicularia small, length to width ratio 1.7–2∶1, monomorphic, attached on a short tubular peduncle at the outer side of proximal gymnocyst of zooids; abfrontal surface of avicularium with only a very shallow indentation at junction between body and rostrum; hooked rostrum, in side view frontal surface is a very shallow S shape, with no peak at mandible hinge. Ovicell spherical, attached at inner distal margin of zooid. Ancrestrula erect, funnel-shaped, without spines.

#### Etymology

From the Latin adjective *albus*, white, in allusion to the whitish colour of the colony that is distinct from the other species of the *B. minima* complex.

#### Remarks

*Bugula alba* n. sp. is characterized by small zooids and avicularia attached at the outer side of the proximal end of zooids. The western Atlantic *B. miniatella*, also has monomorphic avicularia placed at the proximal end of zooids, but differs from *B. alba* n. sp. in having larger zooids (Table 2), a different avicularium shape and a darker colony colour. Another species of this complex occurring in Atlantic waters, *Bugula migottoi* n. sp., is distinct by having a reddish colony and longer avicularia.

#### Distribution (present study)

Brazil: Maceió, AL (Table S1); on algae pelagic *Sargassum* sp.

***Bugula migottoi*** Vieira, Winston & Fehlauer-Ale **n. sp.**

urn:lsid:zoobank.org:act:C3CA5AB0-737A-416B-B20F-91C372C7B697.

(Figures 6B, D, F; Table 2).

**Material examined. Holotype**: MZUSP 601, Ilha do Mel, PR, Brazil. **Paratypes**: MZUSP 602–606, Ilha do Mel, PR, Brazil. **Additional material**: MZUSP 607, Ilha do Mel, PE, Brazil; MZUSP 608–609, Caraguatatuba, SP, Brazil; MZUSP 610–616, Ilha Grande, RJ, Brazil.

#### Type locality

Pontinha, Ilha do Mel, PR, Brazil: 25°33′51′′S, 048°19′00′′W.

#### Diagnosis

Colony translucent brownish red in colour; zooids long (ca. 0.58 mm long and 0.18 mm wide) in two series; elongate avicularia (ca. 0.16 mm long) attached at the outer side of the proximal end of zooids, smaller than those of *B. miniatella*, but longer than those of *B. alba* n. sp.

#### Description

Colony erect and small; living colonies translucent, brownish red in colour. Colonies consist of bifurcating biserial branches, made up of alternating rows of lightly calcified zooids. Zooids elongate, more or less rectangular, slightly broader distally than proximally, with frontal membrane covering almost the entire frontal surface. Distal ends obliquely truncate, rounded, without true spines. Avicularia elongate, length to width ratio 2–2.3∶1, monomorphic, abfrontal surface with a shallow indentation between body and rostrum, attached by a conspicuous peduncle at the proximal end of the outer lateral walls of the zooid; body and rostrum curved, frontal surface profile a very shallow S shape, rostrum sharp hooked distally. Ancrestrula erect, funnel-shaped, without spines, anchored by long stolon-like rhizoids. Ovicells not observed.

#### Etymology

Named after Dr. Alvaro E. Migotto (CEBIMar-USP) for his studies on the marine invertebrate fauna of Brazil, and particularly for having helped us collect and study Brazilian bryozoans.

#### Remarks

This species is similar to *B. alba* n. sp. and *B. miniatella* in having narrow branches and avicularia attached at the proximal ends of zooids. *Bugula alba* n. sp. has a distinct white colour to the colony, smaller zooids and shorter avicularia. *Bugula miniatella* has also a reddish colony, but the avicularia are larger and more elongate (0.19–0.25 mm) than in *B. migottoi* n. sp. (0.15–0.18 mm) (Table 2).

#### Distribution (present study)

Brazil: Angra dos Reis, RJ (Table S1); São Sebastião, SP (Table S1); Ilha do Mel, PR (Table S1); found on algae and stones, in association with other cryptic bryozoans, as well as among hydroids and larger erect bryozoan colonies at 0–20 meters depth.

**Figure 9:**
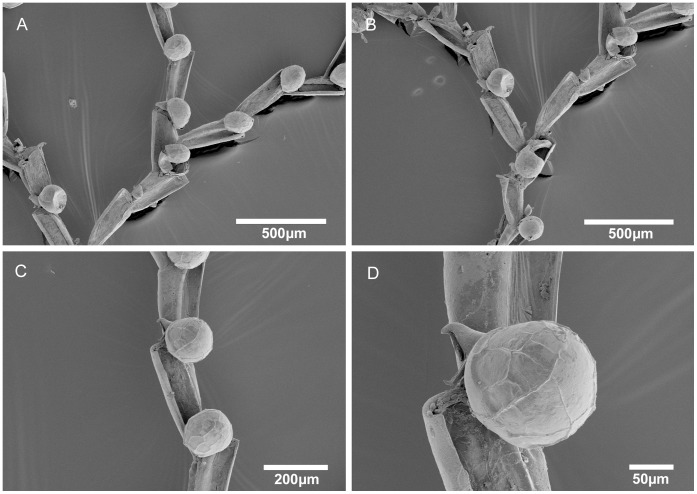
Scanning electron micrographs of *Bugula gnoma* n. sp. *Bugula gnoma* n. sp., MZUSP 623, Paratype, Alagoas, Brazil, (A,B) colony with detail of bifurcation, (C) branch, (D) close-up of ovicelled zooid and avicularium.

### Species of the *Bugula Uniserialis* Group

*Bugula uniserialis* Hincks, 1884 was originally described from Australia [50] (NHMUK 1899.5.1.413), and is characterized by long zooids alternating in two series, bifurcation of type 3 [43], with a slender proximal gymnocyst occupying about 1/3 of zooidal length, and avicularia inserted at the proximal end. At least four morphologically similar species have been described: *Bugula pedunculata* O’Donoghue, 1925 from California [51] (later synonymized under *B. minima* [47]); *Bugula protensa* Hayward, 1981 from deep waters of Gulf of Panama [52]; *Bugula scaphula* Tilbrook, Hayward & Gordon, 2001 from Vanuatu [53]; and *Bugula scaphoides* (Kirkpatrick, 1890), from the Indo-Pacific [54].

Several authors have reported *B. uniserialis* as a circumtropical shallow water species [20], [23], [47], [48], [54], [55], [56]. However, specimens recorded from Atlantic waters are distinct from the Galapagos specimens described by Hastings [48] in the shape of the zooid and the size of the frontal membrane. Morphological variation of both Atlantic and Eastern Pacific specimens from Hinck’s Australian typeled Fransen [57] to consider the Caribbean specimens to be of uncertain status. Recently, the disjunct distribution of *B. uniserialis* was explained by its rarity in collections due to the small and inconspicuous nature of the colonies [23], but specimens from different localities have not been compared in morphological detail or genetically to examine the putative intraspecific variation. Due to such inconsistences in the literature, we examined the zooidal morphology and 16 rDNA sequences of some specimens that had been preliminarily assigned to *B. uniserialis*.

Our specimens from four Brazilian localities show three distinct forms of avicularia (Figures 7A–D): a large (ca. 0.137 mm long and 0.086 mm in diameter), slightly more elongate _avicularium_ with an obtuse peduncle (from São Paulo, Figure 7A); a large (ca. 0.127 mm long and 0.087 mm in diameter) and more compract avicularium (from Rio de Janeiro and Paraná, Figures 7B–C); and a small, very compact avicularium (ca. 0.076 mm long and 0.047 mm in diameter), with short and slender rostrum (from Alagoas, Figure 7D). We have assigned specimens from São Paulo and Alagoas States to two distinct species, *Bugula biota* n. sp. (Figures 7A, 8A–D) and *Bugula gnoma* n. sp. (Figures 7D, 9A–D), respectively.

In spite of the similarities in avicularian morphology between specimens from Rio de Janeiro and Paraná, they present remarkable differences in size (0.115–0.143 mm length for Rio de Janeiro and 0.087–0.115 mm long for Paraná), and in the presence of a long peduncle cushion in colonies from Rio de Janeiro (Figure 7B). These differences led us to describe *Bugula ingens* n. sp. from Rio de Janeiro, and *Bugula rochae* n. sp. from Paraná. Unfortunately, owing to the small size and poor condition of the preserved colonies, it was not possible to ascertain the status of the specimens from Saint Peter and Saint Paul Archipelago assigned by Vieira *et al.* [56] as *Bugula* aff. *uniserialis*.

***Bugula biota*** Vieira, Winston & Fehlauer-Ale **n. sp.**

urn:lsid:zoobank.org:act:2ECBBD17-D55E-4F02-AC8E-B590A10499ED.

(Figures 7A, 8A–D; Table 3).

**Material examined. Holotype**: MZUSP 617, São Sebastião, SP, Brazil. **Paratypes**: MZUSP 618–619, São Sebastião, SP, Brazil. **Additional material**: 620–621, São Sebastião, SP, Brazil.

#### Type locality

Segredo, São Sebastião, SP, Brazil: 23°49′42′′S, 045°25′16′′W.

#### Diagnosis

Erect colonies, translucent yellow to brownish in colour; alternating elongate zooids (ca. 0.77 mm long and 0.13 mm wide) and large avicularia (ca. 0.137 mm long) attached at a short obtuse base at the proximal end of the outer lateral wall of zooid on a short peduncle cushion.

#### Description

Colonies erect, made up of a few flexible, unjointed, translucent yellow to brownish colored branches. Zooids biserial, alternating, but with uniserial appearance, with 3–5 zooids occurring between branch bifurcations. Zooids elongate, tapering proximally, with frontal membranous area occupying distal half of total length. Pedunculate avicularia monomorphic, large (length to width ratio 1.5–1.7∶1), attached to a short obtuse base at the proximal end of the outer lateral walls of zooids on a short peduncle cushion; rounded body with an abfrontal shallow V-shaped indentation between body and rostrum, rostrum short and sharply hooked with a noticeably hooked mandible. Rhizoids attached at the basal part of the zooecia. Ovicells globular, attached in the inner side of the distal end of zooids. Ancestrula erect, funnel-shaped, without spines.

#### Etymology

The species name “biota” is given as a noun in apposition, honoring the ‘Biota-FAPESP Program’ (http://www.fapesp.br/biota/) for funding and promoting the knowledge of Biodiversity in Brazil.

#### Remarks

The examination of Brazilian specimens previously identified as *B. uniserialis*, i.e. colonies with elongate zooids and with a uniserial appearance, revealed four species, distinct from each other by the size of the zooids, the position of the colony on the substratum, the shape and size of the avicularia, and the insertion position of the peduncle bases on zooids. Hastings [48] noted similarities between the Australian specimens attributed to *B. uniserialis* and *B. pedunculata* from southern California (USA) [51], which could be distinguished by the differing size of zooidal membranous area; the Galapagos specimens were also differentiated by zooids with shorter membranous area than those of Australian and Californian colonies [48]. Further studies are required to compare the Brazilian species with specimens previously identified as *B. uniserialis* [47], [48], and results may reveal a higher diversity for this group of *Bugula*, as recently shown for the *B. minima* complex [8]. *Bugula biota* n. sp. is quite distinct from other *uniserialis*-like species by the shape of avicularia with an abfrontal shallow V-shaped indentation (Figure 7A), and by its brownish coloured colonies. This species is similar to *B. uniserialis* reported from the São Paulo by Marcus [20], but his specimens belong to *B. rochae* n. sp. (see below).

#### Distribution (present study)

Brazil: São Sebastião, SP (Table S1); on algae and panels with other bryozoans at 0–3 meters depth.

***Bugula gnoma*** Vieira, Winston & Fehlauer-Ale **n. sp.**

urn:lsid:zoobank.org:act:9A85C6E1-2F9D-401C-9921-4B24E33B6DA1.

(Figures 7D, 9A–D; Table 3).

**Table 3:** Measurements (mm; n = 15) of *Bugula uniserialis* “group” found in Brazilian coast.

	*Bugula gnoma* n. sp.	*Bugula biota* n. sp.	*Bugula ingens* n. sp.	*Bugula rochae* n. sp.
Zooid length				
Range	0.442–0.662	0.690–0.870	0.488–0.644	0.552–0.828
Mean (St. dev.)	0.555 (0.064)	0.776 (0.054)	0.584 (0.051)	0.687 (0.100)
Zooid width				
Range	0.101–0.120	0.120–0.138	0.110–0.129	0.129–0.147
Mean (St. dev.)	0.111 (0.008)	0.132 (0.007)	0.122 (0.006)	0.139 (0.007)
Frontal membrane				
Range	0.276–0.322	0.350–0.478	0.304–0.368	0.276–0.414
Mean (St. dev.)	0.302 (0.016)	0.386 (0.035)	0.329 (0.018)	0.352 (0.052)
Avicularia length				
Range	0.064–0.087	0.129–0.143	0.115–0.143	0.087–0.115
Mean (St. dev.)	0.076 (0.007)	0.137 (0.004)	0.127 (0.009)	0.100 (0.008)
Avicularia width				
Range	0.041–0.051	0.083–0.092	0.074–0.097	0.060–0.074
Mean (St. dev.)	0.047 (0.003)	0.086 (0.003)	0.087 (0.007)	0.066 (0.004)
Ovicell width				
Range	0.129–0.161	–	–	0.138–0.184
Mean (St. dev.)	0.140 (0.010)	–	–	0.161 (0.018)

**Material examined. Holotype**: MZUSP 622, Maceió, AL, Brazil. **Paratypes**: MZUSP 623–625, Maceió, AL, Brazil; UFAL 0214, Maceió, AL, Brazil. **Additional material**: UFAL 048, Marechal Deodoro, AL, Brazil.

#### Type locality

Riacho Doce, Maceió, AL, Brazil: 9°34′42′′S, 035°39′19′′W.

#### Diagnosis

Recumbent colonies, small and translucent white in colour; elongate zooids (ca. 0.55 mm long and 0.11 mm wide) and small avicularia (ca. 0.076 mm long) attached at the proximal end of the outer lateral wall of zooid on a inconspicuous peduncle cushion. Avicularia smaller than those of *B. biota* n. sp., *B. rochae* n. sp. and *B. ingens* n. sp.

#### Description

Colonies recumbent branching, small and translucent, white in colour. Zooids biserial, alternating, but with a uniserial appearance due to slender proximal gymnocystal tubes. Zooids lightly calcified, elongate, about 0.55 mm long, narrower proximally and wider distally, about 0.11 mm wide, with frontal membranous area about 0.30 mm long. Distal ends angled, without spines. Small monomorphic pedunculate avicularia (length to width ratio 1.45–1.7∶1), with only a slight abfrontal indentation, attached at the proximal end of the outer lateral walls of zooids, on a slender peduncle cushion; avicularia sometimes obscured by ovicell of adjacent zooid, with a rounded body and a very short hooked rostrum; mandible strongly hooked. Ovicell globular attached to the inner side of distal end of zooid. Ancrestrula recumbent, shorter than autozooids, without spines.

#### Etymology

From the Latin adjective *gnomus*, dwarf, referring to the small size of avicularia.

#### Remarks

*Bugula gnoma* n. sp. is characterized by elongate zooids with uniserial appearance and very small latero-proximal avicularia, attached by a very short and inconspicuous peduncle cushion. *Bugula gnoma* n. sp. differs from *B. biota* n. sp., *B. rochae* n. sp. and *B. ingens* n. sp. by the presence of recumbent colonies and very small avicularia.

#### Distribution (present study)

Brazil: Maceió and Marechal Deodoro, AL (Table S1); on pelagic *Sargassum* algae.

**Figure 10:**
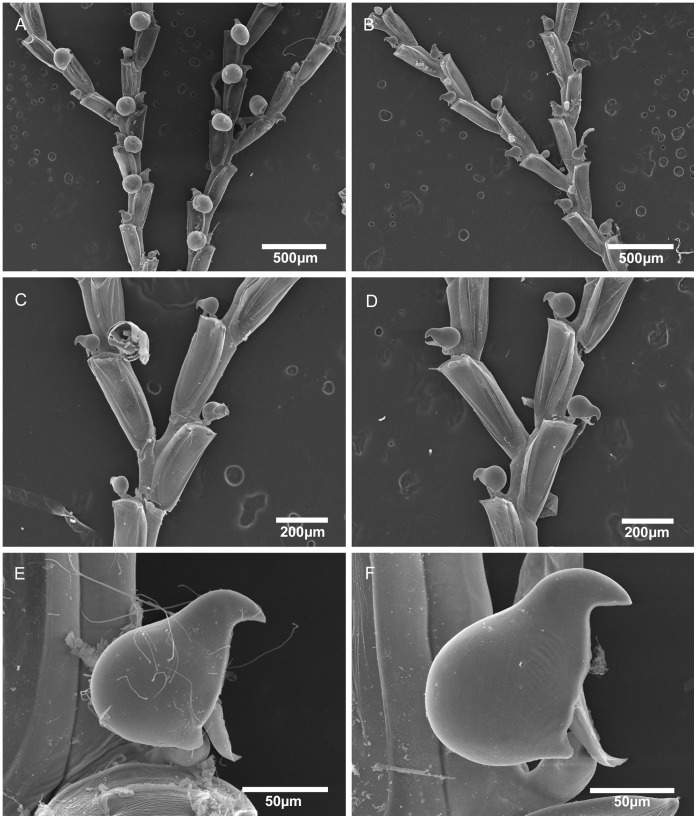
Scanning electron micrographs of *Bugula rochae* n. sp. and *Bugula ingens* n. sp. A– A,C,E, *Bugula rochae* n. sp., MZUSP 631, Paratype, Paraná, Brazil, (A) branches, (C) bifurcation, (E) close-up of avicularium. B,D,F, *Bugula ingens* n. sp., MZUSP 627, Paratype, Rio de Janeiro, Brazil, (B) branches, (D) bifurcation, (F) close-up of avicularium.

***Bugula ingens*** Vieira, Winston & Fehlauer-Ale **n. sp.**

urn:lsid:zoobank.org:act:860B06A0-8132-42DA-B749-689EC4CF0448.

(Figures 7B, 10B, D and F; Table 3).

*?Bugula uniserialis*: Ramalho *et al.* 2005: p. 236, figure 4 [23]. Non *Bugula uniserialis* Hincks, 1884 [50].

**Material examined. Holotype**: MZUSP 626, Ilha Grande, RJ, Brazil. **Paratypes**: MZUSP 627–628, Ilha Grande, RJ, Brazil. **Additional material**: MZUSP 629, Ilha Grande, RJ, Brazil.

#### Type locality

Meros, Ilha Grande, Angra dos Reis, RJ, Brazil: 23°13′S, 044°20′W.

#### Diagnosis

Erect colonies, translucent tan colored; elongate zooids (ca. 0.58 mm long and 0.12 mm wide) and large avicularia (ca. 0.13 mm long) attached at a cuspidate base at the proximal end of the outer lateral wall of zooid on a long peduncle cushion. Avicularia larger than those of *B. gnoma* n. sp. and *B. rochae* n. sp.

#### Description

Colonies erect, usually small, made up of a few flexible, unjointed, transparent tan colored branches. Zooids biserial, alternate, but with uniserial appearance, with 2–5 zooids occurring between branch bifurcations. Zooids lightly calcified, elongate, narrower proximally than distally, with frontal membranous area occupying three fifiths of frontal surface. Large pedunculate avicularia, monomorphic (length to width ratio 1.3–1.55∶1), with a shallow U shaped indention marking the abfrontal surface between body and rostrum, attached at a tubular projection at the proximal end of the outer lateral walls of zooids by short and cuspidate peduncle on a long peduncle cushion; rounded body and short hooked rostrum; mandible hooked, lateral wall in profile showing an irregular edge. Ovicells not observed. Ancestrula recumbent, shorter than autozooids, without spines.

**Figure 11:**
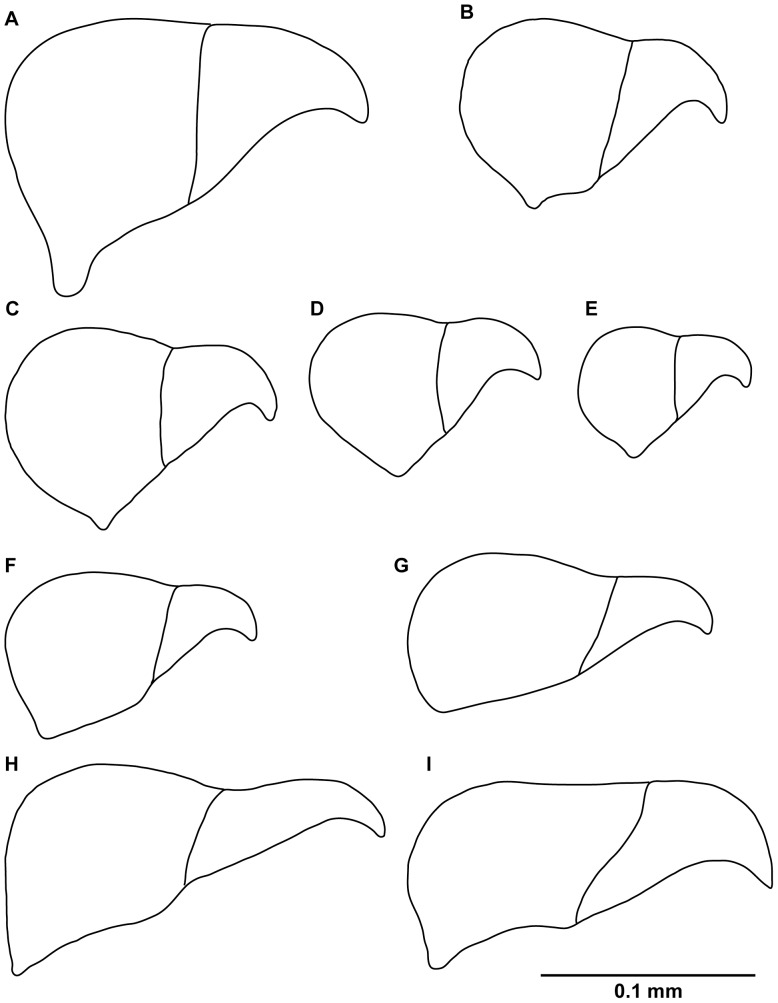
Schematic drawing of avicularia of new Brazilian species of *Bugula*. Schematic drawing of avicularia, (A) *Bugula foliolata* n. sp., (B) *Bugula biota* n. sp., (C) *Bugula ingens* n. sp., (D) *Bugula rochae* n. sp., (E) *Bugula gnoma* n. sp., (F) *Bugula alba* n. sp., (G) *Bugula migottoi* n. sp., (H) *Bugula guara* n. sp., (I) *Bugula bowiei* n. sp.

#### Etymology

From the Latin adjective *ingens*, huge, in allusion to the size of the avicularia.

#### Remarks

*Bugula ingens* n. sp. is quite distinct from *B. gnoma* n. sp. and *B. rochae* n. sp. by means of its conspicuously large avicularia. This species was probably reported under the name *B. uniserialis* from Rio de Janeiro [23], but additional studies on the morphology of avicularia are needed to confirm the identity of these specimens. Marcus [20] reported a similar species from the São Paulo, also under the name *B. uniserialis*, but his specimens (MZUSP 009) belong to *B. rochae* n. sp., distinct by the size of avicularia. The ovicells were neither observed in this study nor by Ramalho *et al.* [23].

#### Distribution (present study)

Brazil: Angra dos Reis, RJ (Table S1); on stones at 5–15 meters depth.

***Bugula rochae*** Vieira, Winston & Fehlauer-Ale **n. sp.**

urn:lsid:zoobank.org:act:6AF5DCD1-8A52-4EC2-96EE-18524AE751AD.

(Figures 7C, 10A, C and E; Table 3).

*Bugula uniserialis*: Marcus, 1937: p. 72, plate 15, figure 38 [14]. Non *Bugula uniserialis* Hincks, 1884 [50].

**Material examined. Holotype**: MZUSP 630, Ilha do Mel, PR, Brazil. **Paratypes**: MZUSP 631–636, Ilha do Mel, PR, Brazil. **Additional material**: MZUSP 637, Ilha do Mel, PR, Brazil; MZUSP 009, *Bugula uniserialis* Hincks, E. Marcus det.

#### Type locality

Ponta da Nhá Pina, Ilha do Mel, PR, Brazil: 25°33′59′′S, 048°18′W.

#### Diagnosis

Erect colonies, translucent yellowish tan in colour; elongate zooids (ca. 0.69 mm long and 0.14 mm wide) and large avicularia (ca. 0.10 mm long) attached at a short cuspidate base at the proximal end of the outer lateral wall of zooid on a small peduncle cushion. Avicularia larger than those of *B. gnoma* n. sp., but smaller than those of *B. ingens* n. sp.

#### Description

Colonies erect, small, translucent yellowish tan in colour. Zooids biserial, alternate, but with uniserial appearance. Zooids lightly calcified, elongate, narrower proximally than distally, with frontal membranous area occupying half of zooidal length. Distal ends angled with sharp points. Small monomorphic pedunculate avicularia (length to width ratio 1.4–1.8∶1) with a rounded body, a shallow U-shaped depression on abfrontal surface between body and rostrum, and short hooked rostrum; mandible hooked at tip; lateral wall in profile a shallow smooth edged S-shaped curve. Avicularia attached at the proximal end of the outer lateral walls of zooids by a short and cuspidate peduncle on a small peduncle cushion. Ovicells globular, attached in the inner side of the distal end of zooid. Ancestrula shorter than autozooids, without spines.

**Table 4:** Valid species of *Bugula* recorded in Brazil.

Species	Occurrence	Depth (m)	Substrata
*Bugula alba* n. sp.	AL	0–2	*Sargassum* sp.
*Bugula biota* n. sp.	SP	0–3	algae and panels
*Bugula bowiei* n. sp.	AL, ES [40], RJ, SP [20], PR [39]	0–20	algae, stones and bryozoans
*Bugula carvalhoi* Marcus, 1949	RJ [23], SP [22]	10	rocks and bryozoans
*Bugula decipiens* Hayward, 1981	PE [62]	943–1007	–
*Bugula dentata* (Lamouroux, 1816)	PE [23], RJ [23], ES [44]	0–15	natural and artifical substrates
*Bugula foliolata* n. sp.	RJ [22], SP [20], [21]	7–20	stones, shells and bryozoans
*Bugula gnoma* n. sp.	AL	0–2	*Sargassum* sp.
*Bugula guara* n. sp.	RJ	5–15	algae, stones and bryozoans
*Bugula ingens* n. sp.	RJ	5–15	stones
*Bugula migottoi* n. sp.	RJ, SP, PR	0–20	algae and stones
*Bugula neritina* (Linnaeus, 1758)	RJ [23], [42], SP [20], PR [39], [63]	0–17	natural and artifical substrates
*Bugula rochae* n. sp.	PR, SP [20]	0–1	stones and bryozoans
*Bugula stolonifera* Ryland, 1960	RJ [23], SP [20], PR [63]	0–10	natural and artifical substrates

#### Etymology

Named after Dr. Rosana Moreira Rocha (UFPR), who has greatly contributed to this work by collecting and donating samples of bryozoans from southern Brazil.

#### Remarks

*Bugula rochae* n. sp. has avicularia about 0.10 mm long, larger than those of *B. gnoma* n. sp, and shorter than those of *B. ingens* n. sp. and *B. biota* n. sp. The insertion of the pedunculate avicularia is distinct in both *B. rochae* n. sp. and *B. ingens* n. sp.; *Bugula ingens* n. sp. has a tubular projection where the peduncule of avicularium is inserted; in *B. rochae* n. sp. the peduncle of the avicularium is attached directly by a very peduncle cushion.

#### Distribution (present study)

Brazil: Ilha do Mel, PR (Table S1); Santos, SP (Table S1); on stones and other bryozoans at 0–1 meter depth.

### Genetic Distance between *B. Ingens* n. sp. and *B. Rochae* n. sp

We obtained sequences of part (∼500 bp long) of the 16 S rDNA gene from eight individuals representing two species of the *B. uniserialis* group, *B. ingens* n. sp. (n = 2), and *B. rochae* n. sp. (n = 6). Each species showed a single unique haplotype (*B. ingens* n. sp., GenBank JQ693390 and BOLD Process ID BRBRY001-12; *B. rochae* n. sp., GenBank JQ693391 and BOLD Process ID BRBRY002-12). We were unable to obtain sequences from *B. biota* n. sp. and *B. gnoma* n. sp. because the only available specimens had been stored in formalin. The sequences from the two new species were 8.8% divergent, a value within the range of the interspecific variation found for the *Bugula* species analysed (3.9% between *B. simplex* and *B. stolonifera* to 22.7% between *B. dentata* and *B. neritina*).

## Discussion

The main morphological features distinguishing among the new species of *Bugula* were found in the position, size and shape of the avicularia (Figures 11A–I). In spite of the importance of SEM morphological studies to distinguish bryozoan species [19], our light microscope images (Figures 7A–D) were also demonstrated to be useful to refine the characterization of the avicularia and to define species boundaries, as in previous taxonomic studies of the genus [8].

Of the species previously reported for the Brazilian coast [58], *Bugula brasiliensis* (d’Orbigny, 1841) from Rio de Janeiro is considered by us here to be a *nomen dubium*, due to the absence of avicularia and ovicells in the original description and illustration [44]. Marcus [20] reported *Bugula ditrupae* Busk, 1858 from São Paulo, but his specimens were assigned to *Bugula aquilirostris* Ryland, 1960, and later synonymized with *B. fulva* [33]; in our study, we showed that *B. fulva* was a misidentification of *B. foliolata* n. sp. Winston [55] reported *B. minima* from Brazilian waters, but no locality or specimens examined were given for this record, which appears to have been an error (J.E. Winston, unpublished data). More recently, two unnamed species of *Bugula* were recorded in the Saint Peter and Saint Paul Archipelago; these resemble *B. uniserialis* and *B. miniatella* [56].

The taxonomic status of the Brazilian specimens attributed to *B. flabellata*, *B. turrita* and *B. uniserialis* are here clarified by detailed studies on zooidal and avicularia morphology. However, further studies on Western Atlantic species, including material from the Caribbean and USA coasts, are needed to solve the identity and the morphological variation among the species reported from those regions, and to compare them with species occurring on the coast of Brazil. Finally, the taxonomic status of *B. dentata*, *B. neritina* and *B. stolonifera* were not reviewed in the present study. Since those species are being reported as cryptogenic across warm and temperate waters around the world [7], [14], [15], we recommend that their nominal validity for the Brazilian specimens receive further examination.

The DNA sequencing aimed to provide an additional tool for distinguishing two morphologically very similar species, *B. ingens* n. sp. and *B. rochae* n. sp. Interespecific 16 S rDNA genetic distances have been also used as complementary identification tools accross distinct bryozoans groups, e.g., Cheilostomata [59], Ctenostomata [60] and Phylactolaemata [61]. Although we do not believe that genetic distances should be used as isolated evidence of differentiation between any particular taxon, we suggest that they may be useful as complementary indicators of the validity of the new species proposed and also in future studies based on DNA barcoding, as already suggested for this segment for other cheilostomes [59].

So far 14 species of *Bugula* are now known from the Brazilian coast [20], [21], [22], [23], [39], [40], [42], [44], [62], [63] (Table 4). This represents a similar species diversity in comparison to those reported in two well sampled localities, viz. the British coast (n = 10) [25] and the SE coast of USA (n = 10) [34]. However, recent studies have shown that hundreds of species of bryozoans may be expected in deep [64], [65], [66] and shallow waters [19], [56], [66], [67], [68] of Brazil. Our results contribute to the knowledge of the species richness of the genus *Bugula* in Brazil, through the description of nine new species, including poorly sampled areas (i.e., northeastern and southern coasts) in addition to the better known southeastern coast. However, we believe that much additional effort is necessary to access more accurately the diversity of this genus in the shallow waters of the Southern Atlantic. Moreover, new sequencing data with from multiple genes need to be generated and extrapolated to species occurring across other seas to be used as complementary tools of taxonomic clarification. Only then, based on a supportive molecular phylogeny in conjunction with homologous morphological characters, will there be a solid basis for a revised taxonomic classification of *Bugula* on a global scale, an effort which will have major implications for our knowledge and ability to manage marine invasive bryozoans, as well.

## Supporting Information

Table S1 **Data of examined material of** ***Bugula*** **.**
(XLS)
